# Recovery Phase Nutrition and Insulin Strategies for a Collegiate Distance Runner with Type 1 Diabetes Mellitus: A Case Study

**DOI:** 10.3390/sports11110214

**Published:** 2023-11-03

**Authors:** Amie E. Schroeder, Richard R. Rosenkranz, Linda K. Yarrow, Mark D. Haub, Sara K. Rosenkranz

**Affiliations:** 1 Department of Food, Nutrition, Dietetics and Health, College of Health and Human Sciences, Kansas State University, Manhattan, KS 66506, USA; richard.rosenkranz@unlv.edu (R.R.R.); lyarrow@k-state.edu (L.K.Y.); haub@k-state.edu (M.D.H.); sara.rosenkranz@unlv.edu (S.K.R.); 2 Department of Kinesiology and Nutrition Sciences, School of Integrated Health Sciences, University of Nevada Las Vegas, Las Vegas, NV 89154, USA

**Keywords:** endurance, athlete, T1D, fueling, carbohydrate supplementation, insulin adjustment

## Abstract

Purpose: There is scant published research regarding nutrition and insulin strategies for athletic performance in collegiate distance runners with type 1 diabetes mellitus (CDRT1). Acute carbohydrate supplementation (CHOsup) and insulin reduction used to minimize hypoglycemia during exercise may result in deteriorated glycemic control post exercise in CDRT1. The present case study of a CDRT1 investigated outcomes associated with a moderate-carbohydrate (ModCHO) diet and 24 h insulin adjustment during recovery phases for improved glycemic control and reduced use of acute strategies. Methods: During an 8-day period, a female CDRT1 followed a ModCHO (~4 g/kg/day) nutrition program. Recovery phase adjustments to insulin doses were made using an equation developed to estimate reduced insulin needs post exercise, as a function of exercise intensity and duration. Daily training was performed in the fasted state at 6:00 a.m. and included additional exercise strategies to reduce glycemic variability when needed. Daily blood glucose time-in-range (TIR) and use of CHOsup were assessed. Athlete well-being was determined using the Student-Athlete Well-Being Scale (SAWS)^TM^ at baseline, and days 1, 3, and 7. Results: Throughout the 8-day period, mean TIR increased (77% versus < 50%) and the magnitude of glycemic excursions decreased (~3.8–15 versus ~3.0–26 mmol/L) relative to a prior comparison period. Minimal pre-exercise CHOsup was employed and CHOsup during exercise was not required. Additionally, the athlete achieved a new lifetime best in the 5000 m run and maintained positive well-being. Conclusion: The present case study provides examples of recovery phase strategies (i.e., ModCHO diet and 24 h insulin adjustments) that may support glycemic control and athletic performance in CDRT1 and provides potential considerations for nutrition and insulin strategies for use by athletes and coaches.

## 1. Introduction

Type 1 diabetes mellitus (T1DM) is an autoimmune disease characterized by an uncontrolled inflammatory response, whereby pancreatic β cells are damaged or destroyed by the immune system causing insulin levels to be severely limited or absent with resultant hyperglycemia [1]. Disease progression necessitates exogenous insulin administration via multiple daily injections (MDI) or continuous subcutaneous insulin infusion [1] (CSII, i.e., pump therapy). The worldwide prevalence of T1DM is 9.5 per 10,000 people, while prevalence in the United States is slightly higher (12.2 per 10,000 people) [2]. Although many people with T1DM compete in sporting events, with some reaching elite or professional levels, the prevalence of T1DM among competitive athletes is currently unknown.

In people without T1DM, euglycemia during exercise is maintained through naturally decreased insulin and increased glucagon to promote increased hepatic glucose production (HGP) in order to match blood glucose (BG) uptake by skeletal muscle [3]. However, people with T1DM are incapable of internal dynamic coordination of metabolic and hormonal responses during exercise, due to lack of endogenous insulin production. When too much exogenous insulin is administered, the circulating insulin–glucagon ratio does not decrease. Consequently, relative “hyperinsulinemia” suppresses HGP, while exercise accelerates skeletal muscle BG uptake, resulting in hypoglycemia (i.e., BG < 3.9 mmol/L).

Accordingly, among current strategies for optimizing athletic performance in T1DM, those aimed at mitigating hypoglycemia during exercise are the most prevalent in published research. However, existing exercise-induced hypoglycemia prevention strategies for athletes with T1DM are primarily acute (i.e., pre- and during exercise), with little attention given to the recovery phases [4] (i.e., periods between workout bouts). Acute approaches for preventing exercise-induced hypoglycemia include carbohydrate supplementation (CHOsup) and insulin reduction strategies. For example, in exercise, ≥60% VO2max sustained for ≥20–60 min, acute carbohydrate (CHO) intake of 75–100 g/h with reduced insulin dose (20–30%) have been suggested [5]. Alternatively, other studies indicate CHO intake of 0.5–1.0 g/kg/h may support euglycemia during exercise [6,7].

Beyond avoiding hypoglycemia during exercise, “late” (i.e., 6–12 h after exercise) and nocturnal hypoglycemia during the exercise recovery phase (i.e., period between training bouts) are also concerns in T1DM. BG can fall markedly > 6 h after exercise [8] due to increased insulin sensitivity that may persist > 24 h post exercise [9]. During the exercise recovery phase, diminished insulin requirements may result in hypoglycemia if appropriate adjustments to insulin doses or CHO intake are not made [8]. Though prevalence of post-exercise hypoglycemia in T1DM is unclear [10], studies suggest that late and nocturnal hypoglycemia are common, especially among children and those performing exercise in the afternoon [11]. Despite the reality of post-exercise hypoglycemia and the potential for increased risk with antecedent hypoglycemic episodes diminishing counterregulatory responses [12], there is little published research indicating specific strategies for mitigating hypoglycemia during the exercise recovery phase, and a “trial and error” approach is generally recommended [4]. Research-based suggestions include reducing bolus insulin by 20–50% with the first meal post exercise, and a similar reduction of basal rate during the first 6–12 h post exercise [4,8,13]. The broad range of insulin reductions suggested by current research may reflect the heterogeneity of post-exercise insulin sensitivity among athletes and raises the question of whether greater specificity can be established to avoid a purely “trial and error” approach. 

Although there is a large body of published research regarding metabolic and hormonal responses to exercise in people with T1DM, evidence-based, sport-specific strategies to support athletic performance in athletes with T1DM across the spectrum of sporting events are lacking. While there are many types of athletes who are not currently represented within published research, the present case study highlights a collegiate distance runner with T1DM (CDRT1) as a unique case from the larger endurance athlete population. We define “collegiate distance runners” as highly trained college athletes competing in 5000 and 10,000 m running events. We distinguish CDRT1 as a distinct subgroup within the broad category of “endurance athlete” because most studies investigating performance strategies for endurance athletes with T1DM involve recreational athletes, marathon runners, or professional cyclists training and competing at intensities and durations that may be different from what 5000 and 10,000 m collegiate runners would typically encounter (Table 1). 

Unfortunately, few extant strategies encompass exercise intensities and durations frequently encountered by CDRT1, necessitating a synthesis of strategies extrapolated from a combination of studies involving runners without diabetes and non-runners with diabetes. Although these acute strategies can be effective for preventing exercise-induced hypoglycemia [5,14], applying commonly used strategies to CDRT1 may lead to deteriorated glycemic control following exercise [15], potentially compromising recovery [16] and glycogen repletion [17], glycemic stability during subsequent training bouts [12], and body composition goals [18].

Given the absence of published research providing specific strategies for glycemic management during the exercise recovery phase, and as an alternative to the acute strategies mentioned above, for the current case study, we used an approach that emphasized the recovery phase. We hypothesized that focusing on recovery phase strategies may be more effective for improving glycemia and athletic performance in CDRT1 than acute strategies alone. The recovery phase strategies included a ModCHO nutrition program and method for calculating reduced insulin needs post exercise based on exercise intensity and duration. In addition to recovery phase approaches, exercise timing and strategies were utilized to further augment glycemic management. Therefore, the aim of the present case study was to evaluate outcomes associated with recovery phase strategies (i.e., ModCHO diet and 24 h insulin adjustments) and exercise timing and strategies, for improving overall glycemia, with reduced reliance on acute strategies, and to support athletic performance in CDRT1. 

## 2. Athlete Background

A collegiate distance runner (i.e., 5000 and 10,000 m), aged 22 years, with a 3-year history of competitive running, diagnosed with T1DM at age 15 years, noted extreme glycemic excursions and chronic pain during their freshman and sophomore years of college running. While CHOsup before and during exercise allowed the athlete to avoid severe hypoglycemia during training and competition, the time between workouts was often marked by a cycle of hypo- and hyperglycemic excursions. 

The athlete and coach questioned whether acute strategies for mitigating hypoglycemia during exercise may be the cause of poor glycemic control throughout the day. Our hypothesis was that prioritizing glycemic control and glycogen repletion during the recovery phase would reduce reliance on acute strategies for hypoglycemia during exercise and thereby improve daily glycemic control, and ultimately athletic performance. During the athlete’s junior year, the athlete and coach partnered to develop recovery phase strategies for daily nutrition, insulin adjustments, and exercise timing, aimed at reversing the cycle of glycemic excursions toward better glucose control and predictability. The following case study is the culmination of their efforts.

## 3. Methods

During an 8-day period, while maintaining the prescheduled training regimen (Table 1), the athlete followed a predetermined daily nutrition program and insulin adjustment strategy, developed in partnership with the coach. The athlete’s training regimen, detailed nutrition program, and insulin adjustment strategy, including rationale, are provided below. 

### 3.1. Nutrition Program

Although a variety of eating patterns can support general health and glycemic management in T1DM [19], this athlete followed a moderate-carbohydrate (ModCHO) diet (i.e., 26–45% total calorie intake) [20], consisting of CHO intake of ~4 g/kg/day (~225 g/day; ~40% total calorie intake) throughout the eight days. While demonstrated to support performance in endurance athletes without diabetes [21], our experience, along with available published research indicate that adherence to a higher CHO diet (i.e., >45–55% total calorie intake or 7–8 g/kg/day) [20] may promote deterioration of glycemic control in T1DM [22,23]. As an alternative, some have considered very low to low CHO diets as a possible strategy for athletic performance and glycemic management in T1DM [24]; however, CHO intakes < 50 g/day may impair exercise economy at higher intensities [25]. Notably, data from a recent survey indicated that > 60% of active individuals with T1DM choose very low to moderate CHO diets [26], shedding light on current practices. With these observations in mind, a ModCHO diet was chosen as a “happy medium” intended to provide the greatest amount of CHO intake feasible without compromising glycemic control. Additionally, consideration was given to choosing a diet that was sustainable in the long term and would not interfere with social interactions for the athlete.

Daily meals were prepared in advance, and macronutrient content was estimated using cronometer.com (Revelstoke, BC, Canada). Three meals were consumed per day (Table 2) at the time of day preferred by the athlete. While a variety of meals (chosen by the athlete) were consumed during the case study, total daily CHO intake was evenly distributed across meals (~70 g/meal) to minimize variability in prandial glycemic response and to simplify glycemic management [19]. Per instruction provided by their diabetes clinic and in keeping with their usual practice, the athlete subtracted total fiber content from CHO for each meal to calculate their bolus insulin dose. However, because this practice is not currently supported by research [19], the fiber content of each meal was consistent for all meals throughout the study to eliminate any potential variation caused by subtracting total fiber from CHO. Protein and fat intakes followed current guidelines for athletes [27] and were spread throughout the day to promote greater predictability of glycemic responses [19]. Moreover, consistent macronutrient distribution allowed for protein and fat insulin requirements to be built in to the athlete’s insulin–carbohydrate ratio (ICR). Additionally, dual-wave boluses (i.e., a combination of an immediate normal bolus and a bolus delivered over a prolonged specified amount of time, referred to as a “square wave”) were employed to account for delayed gastric emptying from fat intake and amino acid gluconeogenesis [28]. An additional consideration was the potential for augmented glycogen synthesis during suboptimal CHO intake with co-ingestion of small amounts of protein [27]. Daily CHO intake was generally low-glycemic load (GL) with high-fiber content, given the evidence supporting general health benefits and glycemic management in T1DM [19], and possible potentiation of insulin activity [26].

In addition to daily meals, a low-CHO, high-protein bedtime snack was consumed once per evening. Although evidence supporting the efficacy of nutritional strategies alone in preventing nocturnal hypoglycemia is lacking in published research [29], this strategy was associated with reduced nocturnal hypoglycemia in the athlete observed in the present case study. When needed, CHOsup was implemented in response to present or imminent hypoglycemia, or when pre-exercise blood glucose (BG) was < 8 mmol/L. Medjool dates (~10 g CHO per date) were ingested when pre-exercise CHOsup was needed (i.e., pre-exercise BG < 8 mmol/L). The athlete and coach found dates favorable because of personal preference for taste, portability, easy dosing of CHO, and a high glucose–fructose content [30]. Though published research is limited regarding the practice of pre-exercise glucose–fructose intake by individuals with T1DM, results from a clinical trial involving 15 men with T1DM indicated that pre-exercise glucose–fructose co-ingestion has a BG preserving effect [31]. During moderate-intensity exercise (i.e., 90 min of cycling at 50% VO2max), a lower incidence of post-exercise hyperglycemia was observed with pre-exercise glucose–fructose co-ingestion compared with glucose intake alone [31]. Bally and colleagues (2017) suggested that this result was due to increased fat oxidation and the glycogen sparing effect of fructose.

### 3.2. Insulin Dose Adjustment

Throughout the study, in alignment with usual practices, the athlete used their personal Medtronic MiniMed 770G System (Medtronic Diabetes, 2022) for blood glucose monitoring (i.e., CGM) and insulin administration (i.e., CSII, pump therapy). It is important to note that although the 770G System has an “auto mode” capability, allowing for the automatic real-time adjustment of basal rate based on the patient’s sensor glucose readings (i.e., “closed-loop system”), the athlete does not normally use auto mode, nor was it enabled during the days of the current study. 

As a means to predict reduced recovery phase insulin needs with greater specificity as compared to a broad range (i.e., 20–50%) currently provided by published research [4], an insulin equation (Table 3) was developed by the athlete and coach with consideration given to research indicating that insulin requirements during the recovery phase may correspond to daily exercise intensity [32]. Development of the equation began with the observation of 24 h trends in the athlete’s glycemic response to various exercise intensities and durations, followed by the creation of an equation (Table 3) designed to calculate a recovery phase insulin dose that matched insulin needs as indicated by the observed trends. During a 6-month period, the equation was tested and modified until calculated daily insulin adjustments produced > 70% BG time in range (TIR; i.e., the amount of time someone with diabetes spends with BG between 3.9 and 10 mmol/L during a 24 h period) during the recovery phase, consistent with the American Diabetes Association guidelines for TIR (i.e., > 70%) [33]. The resultant insulin equation determines the percent reduction of insulin total daily dose (-%TDD) as a function of exercise intensity (% VO2max) and duration, and then uses -%TDD to calculate daily adjustments to temporary basal rate (TBR), insulin–carbohydrate ratio (ICR), and correction factor (CF). Importantly, the constant 0.75 was empirically determined to appropriately reduce the impact of the exercise duration component in the calculation of -%TDD. Trial and error indicated inputting 100% of exercise duration resulted in over or under dosing of insulin at longer and shorter durations, respectively. During the present case study, daily adjustments to TBR, ICR, and CF were manually programmed into the athlete’s pump immediately following completion of daily exercise for the subsequent 24 h period until the next training bout.

It should be noted that the athlete’s %VO2max during exercise was not measured in this case study; rather, estimates of %VO2max at various training paces were determined according to Jack Daniels’ training pace calculator and the American College of Sports Medicine guidelines for aerobic exercise testing and prescription [34,35]. We recognize that expressing relative exercise intensity as %VO2max has been criticized for its failure to account for individual differences in metabolic stress caused by level of fitness and other physiological factors [36]. However, for the purpose of the present case study, we have chosen to define exercise intensity as %VO2max for ease of comparison with related studies and because case study results would not be affected by individual differences. 

No pre-exercise reductions to bolus doses were implemented, as all exercise was performed during the fasted state throughout the 8-day data collection period (see “Exercise Timing” section). Likewise, no planned reductions of pre-exercise basal insulin were implemented, as this practice had been previously observed by the athlete and coach to result in post-exercise hyperglycemia in the athlete. Basal rate suspension during exercise was always employed per the athlete’s preference to be “free” from their pump while running. To account for basal insulin “missed” while running, an equivalent bolus insulin dose was administered when pump therapy resumed immediately following exercise. Notably, post-exercise bolus insulin applied for missed basal insulin during exercise was delivered as a “square wave” (i.e., single bolus delivered evenly over an extended amount of time as specified by the user) over a period equal to the number of minutes that the basal rate was suspended during exercise. To our knowledge, this is a novel strategy, determined solely by the athlete and coach. 

While it is common for people with T1DM to lower their ICR in the morning to account for the apparent increase in insulin requirement for breakfast compared with lunch or dinner (i.e., “dawn phenomenon”) [37], throughout the 8-day data collection period, the athlete’s ICR was not lowered for breakfast as the athlete reported that CGM readings prior to the case study indicated that lowering ICR in the morning resulted in BG trending low around noon. After several practices prior to the case study, the athlete determined that early (i.e., 45 min pre-meal) compared with standard (i.e., 20 min pre-meal) administration of breakfast bolus insulin controlled the prandial glycemic response more effectively. Therefore, although not in accordance with current ADA recommendations, early delivery of breakfast bolus insulin was practiced throughout the duration of the present case study. 

It is important to address the critical nature of establishing appropriate baseline insulin titration before attempting insulin reduction strategies for glycemic management around exercise. In our experience, insulin reduction strategies that are added to a foundation of poorly dosed baseline insulin are problematic. Though it is outside of the scope of the present case study, we hypothesize that programming the correct basal rate may be the key component underpinning effective pump therapy and insulin reduction strategies for exercise. In our experience, when basal insulin rate is poorly matched to HGP, glycemic excursions typically lead to compensatory prandial bolus doses, making an accurate ICR difficult to determine. When ICR is mismatched, glycemia may become a pendulum of excursions swinging between highs and lows. Observations from this athlete and the present case study indicate the establishment of the athlete’s baseline basal rate should be prioritized, followed by ICR titration, and then utilization of insulin reduction strategies for exercise. Several studies support basal rate programming according to circadian rhythms and demonstrate the validity of the so-called “dawn phenomenon” [37,38]. The present case study adds support for such an approach, observing a marked increase in the athlete’s insulin requirement between 3:00 and 7:00 a.m. Though less prevalent, “dusk phenomenon” [38], a secondary less prominent peak in insulin requirement at dusk (i.e., 4:00–7:00 p.m.), has also been noted by the athlete in the present case study. Various methods for self-evaluating individual basal insulin requirements have been suggested, including 24 h fasted tests targeted at detecting periods of inappropriate insulin infusion [38]. Notably, changes in fitness and body weight can influence an athlete’s baseline insulin requirements. Therefore, athletes should be encouraged to work with their Certified Diabetes Care and Education Specialist to periodically reevaluate baseline TDD, basal rate, and ICR.

### 3.3. Exercise Timing and Strategies

Table 1 represents the athlete’s training regimen during the case study. Per usual schedule, the athlete’s training was conducted at 6:00 a.m. every day, with no planned afternoon exercise. This strategy was in alignment with previous studies indicating greater glycemic stability, including reduced late-onset hypoglycemia following morning exercise during the fasted state, as compared with post-prandial or afternoon exercise [39,40]. 

As the pump was not connected to the athlete during exercise, the athlete’s self-perception of glycemia while running determined whether action steps were required to prevent hypoglycemia during exercise. During the 6-month period prior to the present case study, the athlete self-reported a greater tendency toward hypoglycemia during running > 30 min performed at < 60% VO2max. To prevent hypoglycemia during lower-intensity exercise (i.e., < 60% VO2max), the athlete and coach developed a “sprint protocol” in which four 60 m sprints (near-maximal effort), with a 20 s recovery jog between sprints, are employed when the athlete sensed their BG was trending low during exercise. This strategy was created using published research indicating that intermittent sprints during moderate-intensity aerobic exercise may attenuate post-exercise hypoglycemia resultant from sprint activity-induced elevations in catecholamines to promote increased liver glycogenolysis [41]. 

During the present case study, in the event of post-exercise hyperglycemia, the athlete was prescribed a prolonged cool-down; if BG was not within range after the prolonged cool-down, an insulin correction dose was considered, depending on the severity of hyperglycemia and BG directional trend, as indicated by the athlete’s CGM. In keeping with published research regarding reductions in correction boluses post exercise [4,13], correction insulin needs during the study were dosed according to the CF calculated by the insulin equation (Table 3).

### 3.4. Data Collection and Analysis

Electronic copies of the athlete’s continuous glucose monitor (CGM) graphs, an 8-day food record, and training schedule during the 8-day case study were obtained from the athlete and their coach. In addition, CGM graphs from an 8-day period occurring 6 months prior to the case study, as well as BG time in range (TIR; i.e., 3.9–10 mmol/L) values from two 14-day periods during the 18 months prior to the case study, were obtained for comparison. Glycemic control, measured as a percent of TIR, was used to assess the effects of the holistic strategies employed during the case study. In addition, magnitude of glycemic variations during the case study and the prior comparison period were evaluated to provide greater detail regarding daily glycemic control. The athlete’s food record and CGM data were utilized to assess reliance on acute CHOsup and pre-exercise insulin reduction, respectively. Additionally, upon obtaining written and oral informed consent, and on days 1, 3, and 7 of the case study, the athlete completed a Student-Athlete Well-Being Scale (SAWS)^TM^ [42] to assess general well-being throughout the duration of the study. Though sometimes overlooked, we maintain the athlete’s general well-being should never be compromised to obtain short-term performance goals. Although variables influencing glycemia can be tightly regulated to achieve the target BG range (i.e., 3.9–10 mmol/L), the impact of restricted autonomy on general well-being should be considered [43,44]. Additionally, a diet maintaining “perfect” glycemic control may not be sustainable if the athlete’s well-being is negatively impacted. The methods used for optimizing glycemic control in this case study were intended to maintain individuality, choice, freedom, and flexibility within the overall plan structure.

Finally, it should be acknowledged that the coach working in partnership with the athlete to develop the strategies observed here is the graduate student-researcher involved with the present case study. This study was approved by the Institutional Review Board (IRB) of Kansas State University. The athlete was not coerced to participate in or complete the case study. The athlete was participating in typical training practices and engagement with the coach. Per the athlete’s usual routine, in-person athlete–coach check-ins were conducted daily throughout the duration of the 8-day data collection, but these meetings were not a part of the case study as the check-ins pertained to more than glucose regulation. Additionally, the athlete’s RN Certified Diabetes Care and Education Specialist was available throughout the study in the event the athlete had any questions or concerns. 

## 4. Results

### 4.1. Daily Glycemic Control

**Figure 1:**
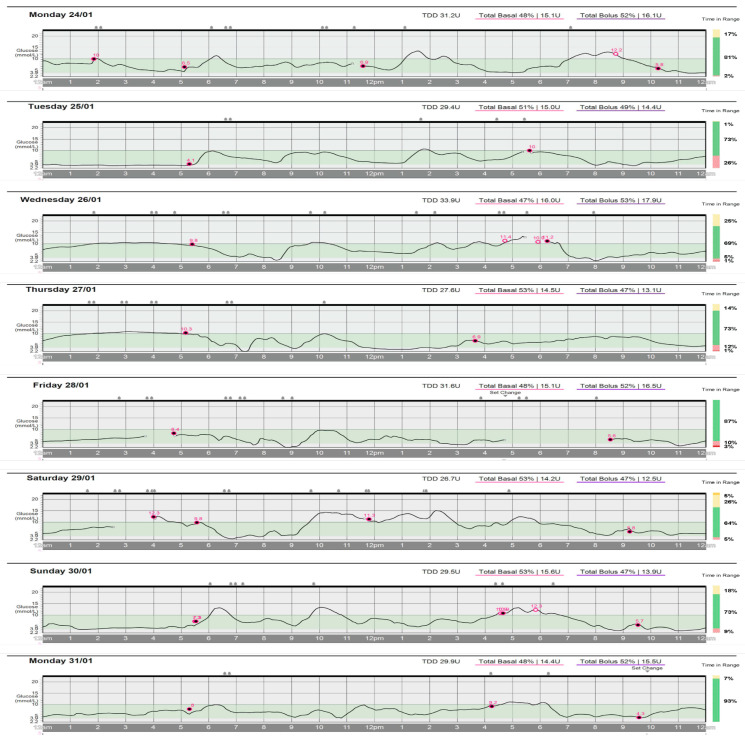
Daily blood glucose data 24–31 January 2022: Graphs depict data captured by the athlete’s continuous blood glucose monitor (CGM) reflecting the athlete’s daily blood glucose 24–31 January 2022; the light gray band represents the target blood glucose range (i.e., 3.9–10 mmol/L). The line break and red circle indicate a period when the athlete was recalibrating their CGM. Abbreviations: TDD = total daily dose, U = units of insulin, ● = GGM sensor calibration.

**Figure 2:**
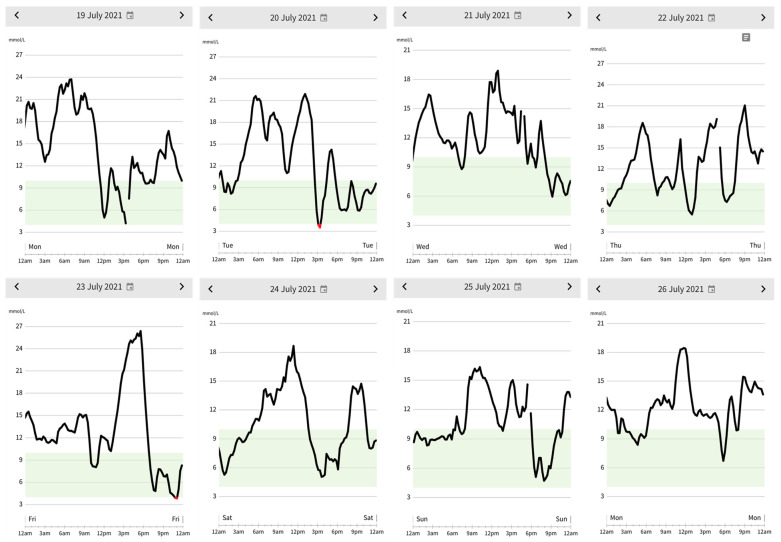
Daily blood glucose data 19–26 July 2021: Graphs depict data captured by the athlete’s continuous blood glucose monitor reflecting the athlete’s daily blood glucose 19–26 July 2021; the light gray band represents the target blood glucose range (i.e., 3.9–10 mmol/L).

Figure 1 and Figure 2 provide a comparison of glycemic control during the present case study (i.e., 24–31 January 2022) and a prior 8-day period (i.e., 19–26 July 2021). As the athlete used a different insulin pump during July 2021 than the pump she used during the current case study, the appearance and details included in the graphs differ. Nevertheless, the figures reveal increased mean TIR (i.e., 77% versus < 50%) and lower magnitude of glycemic variation (i.e., ~3.8–15 versus ~3.0–26 mmol/L) during the current case study compared to the previous 8-day period. 

The athlete’s mean TIR during the current 8-day case study was 77%, which exceeds current ADA guidelines for TIR (i.e., > 70% TIR) [33]. Additionally, CGM data collected from two 14-day periods during the 18 months prior to the case study revealed the athlete’s time in range improved by > 20% during the current 8-day case study compared with each of the 14-day comparison periods (i.e., Jan 2022 TIR = 77% versus Jul 2020 TIR = 56% and Oct 2020 TIR = 51%). Moreover, as Figure 1 and Figure 2 illustrate, in addition to higher TIR, the extent of hyperglycemic excursions was markedly lower during the case study, with BG never rising above 15 mmol/L during the case study compared with multiple departures between 15 and 26 mmol/L observed daily during the prior July 2021 period.

### 4.2. Reliance on Acute Hypoglycemia Prevention Strategies

#### 4.2.1. Acute Carbohydrate Supplementation

CHOsup during exercise was never used during the case study, and pre-exercise CHOsup was only implemented when pre-exercise BG was < 8 mmol/L (i.e., days 1, 2, 7, and 8), and was ingested in relatively small amounts (Table 4; i.e., < 30 g). Limited use of acute CHOsup did not result in hypoglycemia, rather the athlete’s BG remained stable and in range (i.e., 3.9–10 mmol/L) for the duration of all exercise throughout the case study, except for pre- and post-exercise hyperglycemia observed around the athlete’s 5000 m race on Day 6.

#### 4.2.2. Acute Insulin Reduction

No pre-exercise insulin dose reduction (i.e., <2 h pre-exercise) was employed throughout the 8-day data collection period, except before competition on Day 6 per the athlete’s usual race day practice. It should be noted that pre-competition insulin reduction may have contributed to pre- and post-competition hyperglycemia, though other factors, such as competition-related stress, may have influenced the athlete’s BG before and after racing [45]. With the exception of Day 6, the athlete’s BG was preserved throughout all exercise during the case study without acute pre-exercise insulin reduction. 

#### 4.2.3. Athletic Performance

The athlete successfully completed all pre-scheduled training (Table 1) during the case study, including the highest mileage per duration of long runs (i.e., Days 1 and 8) of their running career to date. Additionally, on Day 6 of the current case study, the athlete recorded a new lifetime best for 5000 m on the track. New personal records during training runs and competition indicate the athlete’s fitness likely exceeded previous levels of fitness experienced by the athlete.

### 4.3. Well-Being

Results of the Student-Athlete Well-Being Scale (SAWS)^TM^ [42] confirm that the athlete’s well-being was not compromised during the case study. Additionally, the athlete reported feeling happier and more satisfied with their athletic performance during the 8-day case study than during the previous 6 months. 

### 4.4. Recovery Phase Strategies

In addition to daily glycemic control, reliance on acute hypoglycemia strategies, athletic performance, and well-being, the recovery phase strategies (i.e., ModCHO diet and 24 h insulin adjustments) and exercise strategies themselves are also part of the overall case study results (Table 4). The following paragraphs highlight results from the athlete’s daily nutrition program, 24 h insulin adjustment strategy, and exercise timing and strategies.

### 4.5. Daily Nutrition Program

Table 4 reflects the athlete’s daily CHO intake according to the athlete’s food diary from the 8-day period, estimated using cronometer.com (Revelstoke, British Columbia, Canada). The athlete’s mean daily CHO intake during the current case study was 225 g/day, which may be contrasted with intake < 130 g/day prior (i.e., >6 months) to the case study according to data self-reported by the athlete. 

### 4.6. 24 h Insulin Adjustments

Exercise duration and intensity were used as variables in an equation (Table 3) developed by the athlete and coach to predict appropriate reductions in the athlete’s insulin TDD, which was then used to calculate daily TBR, ICR, and CF. Table 4 reflects the daily TBR, ICR, and CF calculated by the insulin equation and programmed into the athlete’s insulin pump during each day of the 8-day collection period, along with the corresponding daily TIR. As indicated in Table 4, daily TIR exceeded ADA guidelines (i.e., >70%) for all but 2 days (i.e., 69% Day 3 and 64% Day 6) during the case study, with an overall mean TIR above (i.e., 77%) ADA guidelines [33]. 

On Day 6 of the case study, the athlete completed their first race since April 2021, competing in the 5000 m run at 1:30 p.m. Although the lowest %TIR (i.e., 64%) was recorded during the 8-day data collection period, glycemic control was markedly better on Day 6 than on previous competition days (i.e., <40% TIR) according to the athlete’s self-report, and no post-exercise hypoglycemia was observed during the recovery phase. 

### 4.7. Exercise Timing and Strategies

Each day of the 8-day collection period, the athlete initiated daily exercise in the fasted state at 6:00 a.m., including on the day of competition (i.e., Day 6) in which a 40 min easy run (i.e., 60% VO2max) was employed to control for potential GV that may have occurred without their usual morning exercise. On Day 6 of the case study, to minimize the effects of lingering bolus insulin during competition, the athlete’s breakfast bolus was delivered at 7:00 a.m., >5 h before starting their pre-race warm-up (i.e., 1:00 p.m.). Additionally, to account for post-competition hyperglycemia, possibly resultant from a combination of excessive acute insulin reduction and hormonal response to competition (i.e., elevated catecholamine activity) [46], a prolonged cool-down (i.e., 35 min) was successfully implemented to bring the athlete’s BG back into range.

## 5. Discussion

**Figure 3:**
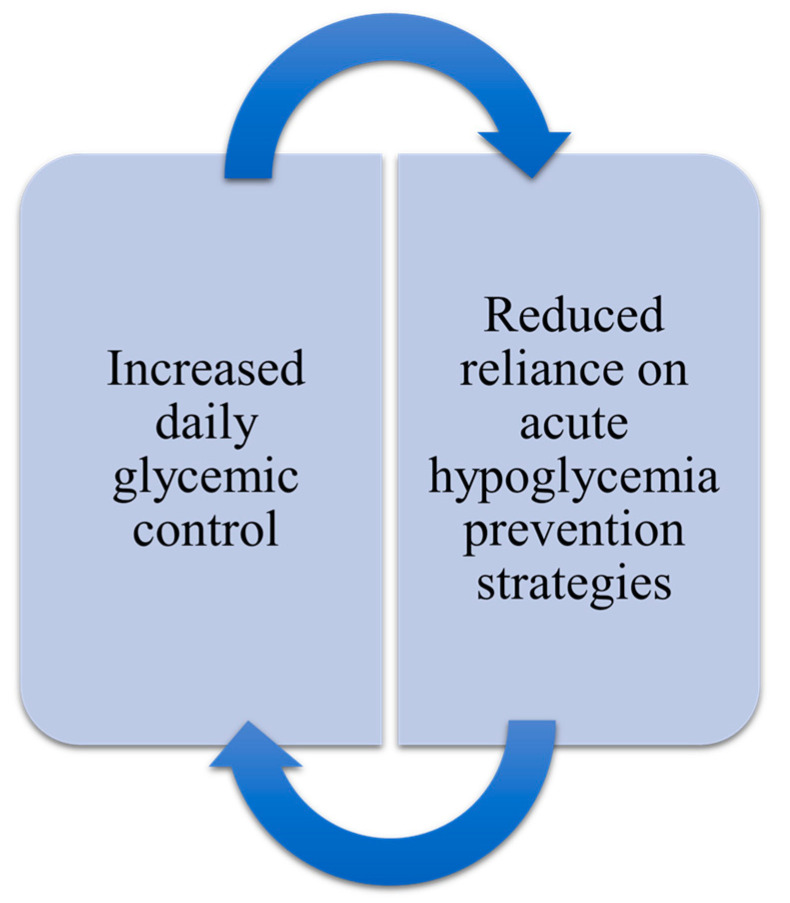
Glycemic stability positive feedback loop: Depiction of the potential cyclic relationship between daily glycemic control (i.e., blood glucose time in range and magnitude of glycemic excursions) and acute (i.e., pre- and during exercise) strategies (i.e., carbohydrate supplementation and insulin reduction) for hypoglycemia prevention during exercise.

The present case study sought to investigate outcomes associated with recovery phase strategies for improving glycemic stability by evaluating the athlete’s daily time in range (TIR) and dependence on acute strategies during the 8-day period. We hypothesized that recovery phase strategies, including a ModCHO diet and 24 h insulin adjustment, and early morning exercise, may be associated with improved overall glycemic control and reduced reliance on acute (i.e., pre- and during-exercise) hypoglycemia strategies (i.e., CHOsup and insulin reduction), thereby promoting a positive feedback loop of improved glycemic stability (Figure 3). 

### 5.1. Daily Glycemic Control

Although hemoglobin A1c (HbA1c, i.e., glycated hemoglobin) is considered the gold standard for assessing diabetes disease development and progression, HbA1c does not provide information regarding acute glycemic excursions and daily trends [47]. Alternatively, TIR provides details about daily glycemic variations and may be a better indicator of overall glycemic control than HbA1c [48]. While current ADA guidelines recommend aiming for > 70% TIR [33], a recent survey of 1026 patients with T1DM revealed that fewer than 20% of respondents perceived their current insulin therapy to be “very successful” in achieving the target TIR [49]. Nonetheless, the results (Table 4) of the present case study demonstrate that it is possible to perform high-intensity aerobic exercise for > 60 min per day while maintaining > 70% TIR. Moreover, results suggest holistic strategies, including a ModCHO diet and 24 h insulin adjustments, and morning exercise, may support meeting TIR guidelines in CDRT1.

It should be noted that on Day 2 of the case study, glucose levels hovered just below 3.9 mmol/L between 12:00 and 5:00 a.m. While this may have been caused by poorly dosed insulin adjustments (i.e., 55% TBR, 1:16 ICR, 3.4 CF) on Day 1 or other uncontrolled variables, we suspect that overcompensating for high BG (i.e., ~12 mmol/L) between 7:30 and 9:00 p.m. on Day 1 (Figure 1) may have resulted in hypoglycemia during the early hours of Day 2. In our experience, and as may have been the case here, fear of hyperglycemia or hypoglycemia may elicit over-compensatory responses to glycemic variation that lead to further deterioration of glycemic control. Nonetheless, the evening of Day 1 provides an example of the numerous decisions required of athletes with T1DM and illustrates the need for more research aimed at developing guidance and systematic approaches for decision making regarding responses to GV, with consideration given to the potential for increased insulin sensitivity post exercise. Future research should consider whether the development of data-informed decision algorithms may promote reduced decision-making burdens, and therefore help to mitigate fear-induced over-compensatory responses. 

### 5.2. Reliance on Acute Hypoglycemia Prevention Strategies

While existent strategies for athletic performance in T1DM are primarily focused on acute (i.e., pre- and during exercise) CHOsup and insulin dose reduction to prevent hypoglycemia during exercise [10], acute strategies may contribute to later deterioration of glycemic control in the athlete. Therefore, we speculated that daily strategies aimed at improving TIR during the recovery phase may increase glycemic stability during exercise and reduce dependence on acute strategies for hypoglycemia prevention.

#### 5.2.1. Acute Carbohydrate Supplementation

In addition to the results reported above, it is notable that during the long runs completed on Days 1 and 8 of the case study, < 30 g CHO was ingested pre-exercise (i.e., ~5:00 a.m.), no CHO was consumed while running, and no acute insulin reductions were made. Importantly, the athlete’s BG was preserved for the duration of both long runs, and late (i.e., 6–12 h post-exercise) hypoglycemia was not observed on either day (i.e., Days 1 and 8). These results may be compared with a randomized controlled trial [41] indicating the greatest risk for exercise-related hypoglycemia may be during or after continuous moderate-intensity (>50% VO2max) exercise, indicating the potential benefits of the holistic strategies applied during the case study in promoting glycemic stability during and after moderate-intensity long-duration exercise. 

#### 5.2.2. Acute Insulin Reduction

Concerning the absence of acute insulin reduction during the case study (except Day 6), we speculate that insulin adjustments made according to exercise duration and intensity facilitated insulin delivery that closely matched post-exercise insulin needs during the recovery phase, providing enough insulin to prevent hyperglycemia, but not in excess requiring acute reduction to avoid hypoglycemia during subsequent exercise. Additionally, a ModCHO diet may have supported glycogen repletion without promoting hyperglycemia, thereby facilitating CHO availability and reducing the need for pre-exercise insulin reduction. Furthermore, in agreement with two randomized crossover trials investigating the effects of morning versus afternoon exercise in people with T1DM [40,50], fasted-state morning exercise may have contributed to greater glycemic stability during training bouts due to the absence of residual bolus insulin on board, eliminating the need for acute insulin reduction before morning exercise [39,40]. 

Although hypoglycemia can be avoided using acute (i.e., pre- and during exercise) CHOsup and insulin reduction strategies, when these strategies are extended to apply to exercise durations and intensities commonly encountered by CDRT1, post-exercise hyperglycemia may occur, presumably because relatively high amounts of CHO intake and insulin reduction are recommended [5,14] to preserve BG during the durations and intensities typical in college-level running. Alternatively, the above results support our hypothesis that recovery phase strategies for glycemic control (i.e., ModCHO diet and 24 h insulin adjustments) and morning exercise may reduce reliance on acute hypoglycemia prevention strategies, promoting a positive feedback loop of improved glycemic stability (Figure 3), as evidenced by the athlete’s mean TIR (i.e., 77%) during the case study. 

### 5.3. Athletic Performance

While many factors may have contributed to the athlete’s overall athletic improvement, we suggest that the holistic strategies employed during the present case study may have promoted improved training and competition performance by supporting increased TIR and reduced reliance on acute hypoglycemia strategies.

### 5.4. Well-Being

Although many uncontrolled factors may have influenced the athlete’s well-being during the case study, improved TIR and reduced magnitude of glycemic excursions requiring treatment may have alleviated stress associated with glycemic variations. Moreover, we speculate that utilization of the daily nutrition program and the insulin equation may have eased the burden of diabetes-related decision making, further promoting the athlete’s well-being during the case study.

### 5.5. Recovery Phase Strategies

#### 5.5.1. Daily Nutrition Program

With consideration given to extant scientific literature, feasibility, sustainability, and the athlete’s preference and experience, a low-GL ModCHO diet was chosen. The athlete’s limited use of acute strategies for exercise-induced hypoglycemia and 77% mean TIR during the case study suggest a low-GL ModCHO diet may support daily glycemic control and reduce reliance on acute strategies for hypoglycemia prevention. Moreover, we speculate that moderate (i.e., 4 g/kg/day) compared with low (i.e., <3 g/kg/day, or <130 g/day) CHO intake may have improved glycogen repletion, potentially improving CHO availability during exercise and reducing reliance on acute hypoglycemia strategies. Additionally, a ModCHO diet, compared with the recommended daily intake of CHO for endurance athletes without diabetes (i.e., 6–10 g/kg/day), may have supported sufficient glycogen repletion without compromising glycemic control. These findings are consistent with a previous randomized controlled trial indicating that a high CHO intake may promote deteriorated glycemic control in people with T1DM [22].

#### 5.5.2. 24 h Insulin Adjustments

We speculated that calculating daily adjustments in insulin TDD as a function of exercise duration and intensity would provide more accurate estimates of daily insulin requirements than the broad range (i.e., 20–50%) currently provided by published research [4]. Results of the present case study indicate insulin adjustments according to daily exercise intensity and duration can support glycemic control during training for college-level distance running. In addition, according to the athlete’s self-report, glycemic control on race day (i.e., Day 6) improved compared with previous competition days (i.e., 64% versus < 40% TIR), suggesting insulin adjustments according to daily exercise intensity and duration may also support improved glycemic stability on race days. While pre- and post-race hyperglycemia occurred, possibly due to competition-related stress, remarkably, no post-exercise hypoglycemia was observed during the recovery phase on Day 6, indicating the potential for insulin adjustments based on exercise intensity and duration to mitigate late and nocturnal hypoglycemia in CDRT1 following the afternoon competition. 

#### 5.5.3. Exercise Timing and Strategies

Consistent with case study findings, we maintain that morning exercise during the fasted state may support glycemic stability during and after exercise. When morning exercise is not possible, initiating pre-exercise mealtime bolus insulin > 5 h before exercise may minimize the effects of lingering bolus insulin during exercise, as demonstrated on Day 6 of the case study. Additionally, in agreement with current research, results from the present case study support the use of a prolonged cool-down to successfully bring the athlete’s BG back into range.

### 5.6. Clinical Implications

**Figure 4:**
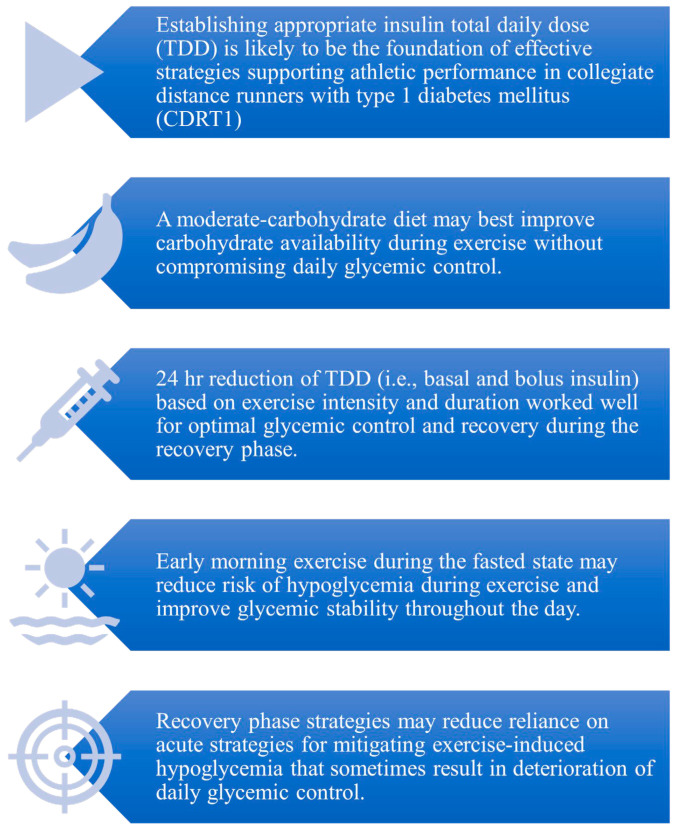
Case study clinical implications: Clinical implications from an 8-day case study in which a collegiate distance runner with type 1 diabetes mellitus utilized recovery phase (i.e., period between workout bouts) strategies for glycemic control, including a moderate-carbohydrate nutrition program, daily 24 h insulin adjustments based on exercise intensity and duration, and early morning exercise regimen.

**Figure 5:**
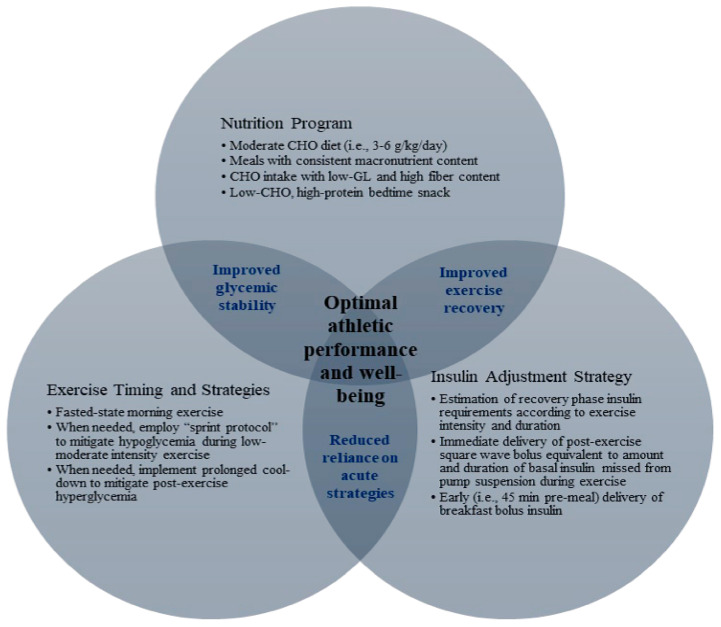
Recovery phase strategies for athletic performance and well-being. Venn diagram depicting how recovery phase (i.e., period between workout bouts) nutrition, insulin, and exercise strategies may improve glycemic stability and exercise recovery, while minimizing acute strategies for hypoglycemia prevention, thereby supporting optimal athletic performance and well-being in a collegiate distance runner with type 1 diabetes mellitus.

As with any case study, there are clear limitations regarding the generalizability of any of the outcomes. Nonetheless, the strategies provided herein, and the equations developed during the course of the case study, may benefit other collegiate endurance athletes and coaches by providing potential considerations for daily nutrition programming, insulin TDD reduction calculations, and strategies for exercise timing. While these strategies will require further research in order to determine generalizability, Figure 4 highlights several possible implications that may be drawn from the present case study, while Figure 5 summarizes the case study strategies. Perhaps most notably, our experience, together with the limited available scientific literature, indicates that adjustments to insulin TDD based on intensity and duration of exercise may support improved glycemic control and recovery in athletes with T1DM. While more research is needed to establish the relationship between various intensities and durations of exercise and reduced insulin requirements post exercise, the present case study provides a promising starting point for future research.

### 5.7. Case Study Limitations

The use of VO2max estimates based on the athlete’s pace per mile to calculate insulin reductions using the insulin equation is an obvious limitation of the present case study. We acknowledge that laboratory measurements of VO2max and %VO2max would provide a clearer assessment of insulin adjustments (i.e., TBR, ICR, and CF) calculated by the insulin equation. However, estimates based on field tests, such as the one used in the current case study, may be valuable for athletes without access to laboratory assessments, or to more closely mimic race conditions. However, population studies using laboratory measurements of VO2max to develop advanced algorithms for automated insulin pump delivery or manual calculations for daily insulin adjustments may be a potential opportunity for future research. The comparison of data (i.e., CGM graphs and TIR) collected during the current case study with previously collected data from periods where a different CGM and insulin pump were used presents another potential limitation of the present case study. While differences in technology may have partially explained differences in glucose outcomes, other case study outcomes, including improved athletic performance and positive well-being, suggest negligible influence of CGM/insulin pump differences on current results. Finally, it should be noted that due to the athlete choosing to be disconnected from their insulin pump while exercising, the athlete’s precise BG during exercise throughout the case study is unknown. Moreover, BG in Figure 1 at the time of exercise may not be accurate, as the athlete’s BG sensor may have been beyond the necessary proximity of their insulin pump for accurate data capture. Nonetheless, based on BG measurements before and after exercise and athlete-perceived glycemia during exercise, it appears that the athlete’s BG remained in a safe range during all exercise performed throughout the case study.

## 6. Conclusions

The present case study demonstrates the potential for a distance runner (i.e., 5000 and 10,000 m) with T1DM to successfully train and compete at the collegiate level without compromising glycemic control and general well-being, and provides considerations for athletic performance in CDRT1. The strategies outlined in the present case study may offer a beneficial alternative to existent acute strategies for athletes with T1DM by potentially supporting increased TIR and reduced reliance on acute strategies for hypoglycemia prevention. Holistic strategies that emphasize the recovery phase, including consuming a low-GL ModCHO diet, 24 h insulin dose adjustment based on exercise duration and intensity, and fasted-state morning exercise, may improve glycemia during exercise and the recovery phase. Together, these strategies may ultimately improve athletic performance and promote general well-being in CDRT1.

## Figures and Tables

**Table 1:** Athlete’s training regimen during case study.

Day	ED (min)	EI (%VO2max)	Description
1	100	60%	long run
2	60	65%	easy run
3	60	60%	easy run
4	75	55%, 75%	tempo run ^a^
5	60	55%, 85%	light pre-race workout ^b^
6	105	60%, 45%, 85%	morning easy run, afternoon race ^c^
7	50	50%	recovery jog
8	100	> 60%	long run

VO2max estimated according to minutes per mile running pace recorded by the athlete’s GPS running watch. Abbreviations: ED = exercise duration, EI = exercise intensity. ^a^ 20 min warm-up/cool- down (55% VO2max), 55 min tempo run (75% VO2max). ^b^ 50 min run (55% VO2max), strides (85% VO2max). ^c^ 40 min morning run (60% VO2max), 45 min warm-up/cool-down (45% VO2max), ~20 min race (85% VO2max).

**Table 2:** Moderate-carbohydrate nutrition program.

	Carbohydrate ^a^	Protein ^b^	Fat ^c^
Macronutrient Servings	1/3 cup oats, dry	1 ounce poultry or beef	2 TBSP nuts/seeds
	1/3 cup brown rice, cooked	1 ounce fish	1 TBSP nut/seed butter
	1/2 cup legumes, cooked	1 large egg	1 TBSP butter
	1/2 cup quinoa, cooked	1 cup milk, 1%	1 TBSP olive oil
	1 cup milk, 1%	1 cup chocolate milk, 1%	1/2 avocado, medium
	1/2 cup chocolate milk, 1%	1/3 cup cottage cheese, 1%	
	1/2 potato, medium	1/3 cup Greek yogurt, 1%	
	1/2 sweet potato, medium	1/4 cup feta crumbles	
	1/4 cup low-fat granola	1/4 cup shredded cheese	
	1 cup berries	1 ounce sliced cheese	
	1/2 piece of fruit		
	2 TBSP dried fruit		
	1 medjool date		
	1 TBSP honey or maple syrup		
	Non-starchy vegetables		
	2 cups leafy greens		
	1 cup raw vegetables		

Daily intake included 3 meals/day according to number of macronutrient servings specified below with 1 serving of non-starchy vegetables/meal; 1 low-carbohydrate (i.e., 10 g), high-protein (i.e., 20 g) bedtime snack/day. ^a^ 5 servings/meal (~70 g). ^b^ 4 servings/meal (~35 g). ^c^ ~1 serving/meal (~10 g).

**Table 3:** Insulin equation.

	Calculation	Example ^a^
−%TDD	[%VO2max × (0.75 ^b^ × ED)] ÷ 100 = -%TDD	[0.65 × (0.75 × 60)] ÷ 100 = 0.29 (i.e., −29%)
TBR	1 − (-%TDD) = TBR	1 − 0.29 = 0.71 (i.e., **71%**)
ICR	450 ^c^ ÷ (TDD_B_ × TBR) = ICR consequent ^d^	450 ÷ (50 × 0.71) = 12.67 (i.e., **1:13**)
CF	94 ^e^ ÷ (TDD_B_ × TBR) = CF	94 ÷ (50 × 0.71) = 2.65 (i.e., **2.7**)

The insulin equation expresses daily insulin adjustments (**bold**) in the form in which they are programmed into the 770G System insulin pump; TBR (percent), ICR (ratio), and CF (factor). Clinical constants derived from Cornell et al. (2021) and King and Armstrong (2007). Abbreviations: ED = exercise duration, -%TDD = percent reduction of total daily dose, TDD_B_ = baseline (i.e., without exercise) total daily dose, TBR = temporary basal rate, ICR = insulin–carbohydrate ratio, CF = correction factor. ^a^ Example of calculations for 60 min run at 65% VO2max; TDD_B_ = 50. ^b^ 0.75 = empirically established constant. ^c^ 450 = clinical constant used to calculate ICR (i.e., 450 ÷ TDD_B_ = ICR consequent). ^d^ Where ratio is a:b, consequent = b. ^e^ 94 = clinical constant used to calculate CF (i.e., 94 ÷ TDD_B_ = CF).

**Table 4:** Case study results and compiled data.

	Carbohydrate Intake (g)			Exercise		Insulin Adjustments			
Day	Daily	Pre-Exercise	During Exercise	Duration (min)	%VO2max	TBR	ICR	CF	%TIR
1	240	30	-	100	60	55	1:16	3.4	81
2	240	30	-	60	65	65	1:13	2.7	73
3	225	-	-	60	60	70	1:13	2.6	69
4	175	-	-	75	55, 75	55	1:15	3.2	73
5	230	-	-	60	55, 85	70	1:12	2.6	87
6	210	-	-	105	60, 45, 85	60, 55	1:16	3.4	64
7	240	20	-	50	50	70, 80	1:13	2.7	73
8	240	20	-	100	>60	55	1:16	3.4	93
Mean	225	12.5	0	~75	>60				~77

VO2max estimated according to minutes per mile running pace recorded by the athlete’s GPS running watch. TBR, ICR, and CF reflect estimates calculated by insulin equation (Table 3); TBR is rounded to the nearest 5% and ICR consequent ^a^ is rounded to the nearest whole number per 770 G insulin pump TBR and ICR setting requirements. Days 6 and 7 each used two different TBRs to account for an afternoon competition on Day 6 (lowest TBR was used to calculate ICR and CF each day). Abbreviations: CF = correction factor, CHO = carbohydrate, ICR = insulin-carbohydrate ratio, TBR = temporary basal rate, TIR = time in range. ^a^ Where ratio is a:b, consequent = b.

## Data Availability

Not applicable.
